# Correlation of serum electrolytes with serial miRNA in advanced stage non-small cell lung cancer (NSCLC) in Indonesia

**DOI:** 10.1186/s13104-021-05852-w

**Published:** 2021-11-27

**Authors:** Arif Riswahyudi Hanafi, Achmad Mulawarman Jayusman, Priscillia Imelda, Serafim Alfasunu, Ahmad Hamim Sadewa, Dibyo Pramono, Didik Setyo Heriyanto, Sofia Mubarika Haryana, Siti Boedina Kresno

**Affiliations:** 1 Department of Pulmonology, Dharmais Cancer Hospital, Jakarta, Indonesia; 2 Lung Cancer Research Team, Department of Pulmonology, Dharmais Cancer Hospital, Jakarta, Indonesia; 3 Faculty of Medicine, Public Health, and Nursing, Universitas Gadjah Mada, Yogyakarta, Indonesia; 4 Department of Clinical Pathology, Dharmais Cancer Hospital, Jakarta, Indonesia

**Keywords:** Advanced stage NSCLC, Potassium, miRNA

## Abstract

**Objective:**

This study aims to evaluate the correlation between electrolytes and serial miRNAs from our previous study. We want to prove that there is the molecular basis that underlying electrolytes disturbances as the predictive indicator to the outcome in NSCLC patients.

**Results:**

There were positive correlation between potassium level with miR-34 (p  = 0.008, r  = 0.366), miR-148 (p  = 0.004, r  = 0.394) and miR-155 (p  = 0.031, r  = 0.300).

## Introduction

Lung cancer is the most common cause of cancer-related death among men in the world [1, 2]. According to data from Dharmais National Cancer Hospital, lung cancer takes first place among others cancer for causing death in men and non-small cell carcinoma lung cancer (NSCLC) constitutes 85% of all lung cancer cases. This high mortality results from the advanced stage as well as the resistance to therapy [3, 4].

Since the very high mortality caused by lung cancer, more precision and sensitive therapy among non-small cell lung carcinoma (NSCLC) patients is needed. There is non-coding small RNA, called micro RNA (miRNA), that play the role to decrease the protein expression of the targeted gene by repressing the translation process or degrading messenger RNA (mRNA) [5]. More than two thousand types of miRNA have been found in humans that target at least 60% mRNA [6]. Our previous study showed that several miRNA expressions, such as miR-34, miR-222, miR-148, and miR-155, have predictive roles in lung cancer, especially NSCLC [7].

Cancer patients often experience abnormalities in the serum electrolytes. The electrolyte disturbance was suggested as the effect of the cancer process itself, called the paraneoplastic process [8]. This is one of the signs related to the poor outcome since the altered electrolyte could be worsening the patient condition. Early recognition and proper correction of abnormal electrolytes are very important in cancer management.

Both miRNA and electrolytes have an important role to show us the outcome of cancer patients. Therefore, we consider continuing our previous study to evaluate the correlation between electrolytes and serial miRNAs to prove that there is the molecular basis that underlying electrolytes disturbances in NCLC patients. We hope that abnormal findings of both indicators should be anticipated to the poor outcome in lung cancer.

## Main text

### Materials and methods

#### Study design and participant

This study was a cohort retrospective study. About 52 patients that meet inclusion criteria from our previous study were included [7].

#### Sample collection

The patient’s blood was drawn with standard procedures [7] to obtained serum and examined both electrolytes and miRNA.

#### miRNA testing

This study was a continuation of previous publication [7]. Target gene transcripts are amplified using specific primers. Amplification using a 7500 Fast (Applied Biosystem) machine with the following steps: (1) initial denaturation at 95 °C for 10 min, followed by 45 denaturation cycles at 95 °C for 10 s and 60 °C for 1 min; (2) melt-curve analysis is performed after 45 qPCR cycles are completed; (3) the amplification results are published in the form of cycle threshold (CT), which is the number of amplification cycles at the time the amplicon is determined to reach the detection threshold (threshold); (4) the level of miRNA expression is expressed as the level of absolute expression and relative expression (fold change). Relative expression is decrement of CT miRNA achieved compared to endogenous control (ΔCT). In this study miR-16 was used as a reference. Fold change is calculated by the formula: 2^−ΔΔCT^ and expressed without units while for the fold regulation value is calculated by calculating the inverse negative of the fold change number previously calculated. So for fold regulation is calculated by the formula fold regulation  = − 1/(fold change).

#### Statistical analysis

Data were analyzed using Mann Whitney-U test for two types of variables or Kruskal-Wallis for more than two types of variables. This study used a significance p value  < 0.05 and a confidence interval of 95%.

### Results

In our previous study, we have presented the demographic data of patients which is illustrated in Table 1 [7]. The incidence of lung cancer was dominated by men (71.2%) with mean ages 55.7 years old. There was a slight difference between non-active smokers (53.8%) and active smokers (46.2%). About half of the subjects were end-stage lung cancer (55.8%) with the most common type of lung cancer was adenocarcinoma (82.7%). Patients in this study mostly have normal serum electrolytes levels, which are sodium (63.5%), potassium (88.5%), chloride (80.8%), and calcium (80.8%). Generally, low levels of serum electrolytes were more common than high levels. There were 18 patients with hyponatremia (34.6%), 5 patients with hypokalemia (9.6%), 9 patients with hypochloremia (17.3%) and hypocalcemia (13.5%).

**Table 1:** Demographic characteristic of advanced stage NSCLC patients in Dharmais Cancer Hospital in 2017

Variable	Number (N)	Percentage (%)	p value
Sex			0.002
Male	37	71.2	
Female	15	28.8	
Age (years)	55.87 ± 11.465		0.928
Mean	55.87 years		
Range	23–88 years		
Age group			0.000
< 40 years	4	7.7	
40–60 years	28	53.8	
> 60 years	20	38.5	
Smoking			0.579
Yes	24	46.2	
No	28	53.8	
TNM stage			0.000
Ivb	29	55.8	
Iva	20	38.5	
IIIb	3	5.8	
Type of cancer cell			0.000
Adenocarcinoma	43	82.7	
Squamous cell carcinoma	9	17.3	
Electrolyte			
Sodium (mmol/L)	135.4 ± 5.77		0.001
> Normal	1	1.9	
Normal (135–150 mmol/L)	33	63.5	
< Normal	18	34.6	
Potassium (mmol/L)	4.25 ± 0.568		0.517
> Normal	1	1.9	
Normal (3.5–5.3 mmol/L)	46	88.5	
< Normal	5	9.6	
Chloride (mmol/L)	98.09 ± 7.57		0.247
> Normal	1	1.9	
Normal (95–111 mmol/L)	42	80.8	
< Normal	9	17.3	
Calcium (mmol/L)	9.2 ± 1.78		0.001
> Normal	3	5.8	
Normal (8.1–10.4 mmol/L)	42	80.8	
< Normal	9	13.5	

The correlation of serum electrolytes with serial miRNA (miR-34, mir-148, miR-222, and miR-155) was showed in Table 2. The results showed no significant correlation between most of electrolyte variables (sodium, chloride, and calcium) with serial miRNAs, except for potassium or kalium which has significant positive correlation with miR-34 (0.008; r 0.366), miR-148 (0.004; r 0.394) and miR-155 (0.031; 0.3). Sodium has p-value 0.312 with miR-34; 0.159 with miR-148, 0.208 with miR-222; and 0.086 with miR-155. Chloride has p-value 0.494 with miR-34; 0.850 with miR-148; 0.426 with miR-222, and 0.365 with miR-155. While calcium has p-value 0.999 with miR-34, 0.525 with miR-148; 0.697 with miR-222; and 0.646 with miR-155.

**Table 2:** Serum electrolytes correlation with serial miRNA of advanced stage NSCLC patients in Dharmais Cancer Hospital in 2017

Electrolyte variables	p value; coefficient of correlation (r)			
	miR-34	miR-148	miR-222	miR-155
Sodium	0.312; − 0.143	0.159; − 0.198	0.208; − 0.177	0.086; − 0.241
Potassium	**0.008**; **0.366**	**0.004**; **0.394**	0.162; 0.197	**0.031**; **0.300**
Chloride	0.494; − 0.097	0.850; − 0.027	0.426; − 0.113	0.365; − 0.128
Calcium	0.999; 0.0002	0.525; − 0.090	0.697; − 0.055	0.646; − 0.065

Continous variables were presented in mean ± standard deviation. TNM stage: notation system that describes the stage of a cancer using alphanumeric codes

### Discussion

Lung cancer still has a high mortality rate in the world. Recent data on our hospital (2003–2007) showed that the mortality of lung cancer was the highest among other cancer and the incidence of lung cancer in men was more common than in women [9]. Those data were the same as the result of this study, where the number of lung cancer was higher in men than women. Smoking is still contributing to the prevalence of lung cancer in this study [1–3]. The number of cigarettes smoked per day has been related to the increasing in lung cancer incidence [10]. Moreover, passive smokers are also being the risk factor for lung cancer, with a high relative risk (RR  = 1.24) [11–15]. However, our study showed slight differences between active smokers and non-active smokers. Most of the patients were adults with a mean age of 55.8 years old. The age was varied among cancer patients, but the youngest and the oldest people will be susceptible, especially to the smoking exposure and the progression to be the lung cancer itself. In Dharmais Cancer Hospital, the most finding type of lung cancer was non-small cell lung carcinoma (NSCLC), with adenocarcinoma type dominated toward squamous cell carcinoma. Generally, the patient who visits Dharmais Cancer Hospital were patients with advanced stages since our hospital was the national reference hospital in Indonesia.

In our study, we find that most of the patients have normal serum electrolytes levels. However, the incidence of low-level serum electrolytes was more common than high-level serum electrolytes. Abnormalities in serum electrolytes can be correlated with inadequate dietary intake in cancer patients. In a cancer patient, electrolyte disturbance was also suggested as the effect of the cancer process itself, called the paraneoplastic process [8]. Nevertheless, patients with comorbidities, renal dysfunction, and the use of anti-tumour therapy have a higher risk for electrolytes disturbance [16]. Hyponatremia and hypokalemia were the most common findings among cancer patients, mostly in advanced cancer, although they can also occur due to other conditions. The previous study considered that hyponatremia in cancer might be the consequence of water retention, continuous loss of sodium, or hypo-osmolality, and it was believed that has an association with the syndrome of inappropriate secretion of anti-diuretic hormone (SIADH) [17]. Meanwhile, hypokalemia was suggested due to Cushing’s syndrome caused by ectopic adrenocorticotropic hormone (ACTH) production by tumours [18, 19]. Hypernatremia was rarely occurring in cancer patients, but hyperkalemia in cancer patients may become one of the important signs for an oncology emergency, called tumour lysis syndrome (TLS) [20]. It is characterized by severe metabolic derangement caused by a rapidly abundant breakdown of tumour cells that release their intracellular contents to the systemic circulation [20, 21]. The risk of TLS will increase in patients that are exposed to chemotherapy, radiation, corticosteroids, or may occur spontaneously. In some cases, hyperkalemia in TLS could be worsening and it causes arrhythmia to death [21, 22]. Prompt recognition and management were needed to prevent poor outcomes.

Our study obtained statistically significant results for the correlation of serum potassium with miR-34 (p  =  0.008, r  =  0.366), miR-148 (p  =  0.004, r  = 0.394) and miR-155 (p  = 0.031, r  = 0.300) which is directly proportional to moderate correlation (r  ≥ 0.3). We suggested that there was a molecular mechanism involved in electrolytes abnormalities of the cancer patient, especially for the increase of potassium or kalium linked with the decreasing of suppressor miRs expression. The correlation of hyperkalemia to the oncomiR expression seems inconclusive, regarding it has no significant result to another oncomiR in our study (miR-222). There is no strong evidence to support the result and the underlying mechanism is still not fully understood. Another study has also found there was a molecular association between hyperkalemia and lung squamous cell carcinoma as the result of RNA expression database examination [23]. However, our study investigates both adenocarcinoma and squamous cell carcinoma and these findings could be said to be novel among lung cancer because there has not been done previously on the relationship between increased serum miRNA with the occurrence of electrolyte disturbances in advanced stage NSCLC patients. One important point is that hyperkalemia in cancer patients, especially advanced-stage NSCLC patients, should be considered as a sign of the possibility of TLS. The patient could be worsening and run to arrhythmia or even death if immediate recognition was ignored.

In conclusion, our study has proved the correlation of electrolytes disturbances, particularly for hyperkalemia, to the serial miRNA, both suppressor miRs and oncomiRs. This study suggests that molecular basis is responsible for electrolytes interference among NSCLC patients. The expression of serial miRNAs in lung cancer could be an early prediction of hyperkalemia. Still, more research should carry out to verify this suggestion. Additionally, our study could consider as the basis for the management of lung cancer itself, regarding hyperkalemia should be anticipated from a life-threatening condition in cancer patients. Perhaps, our findings may be valuable for promising molecular treatment in the future to improve the life expectancy of lung cancer patients.

## Limitation

Our study was not examining other laboratory indicators for TLS, such as uric acid, creatinine, and phosphoric acid. Further study should be done to prove this suggestion.

## Data Availability

The datasets generated and/or analyzed during this study are restricted to publicly share due to some datasets used are extracted from our previous published study (https://doi.org/10.3779/j.issn.1009-3419.2020.104.02) and some important datas are available in database of Dharmais Cancer Hospital. Data are however available from the corresponding author upon reasonable request and permission of Dharmais Cancer Hospital.

## Abbreviations

NSCLC: Non-small cell lung carcinoma
miRNA: Micro RNA
mRNA: Messenger RNA
SIADH: Secretion of anti-diuretic hormone
ACTH: Adrenocorticotropic hormone
TLS: Tumor lysis syndrome
